# From Local Crafts to Market Niches: The Marketing Potential of Artisanal Foods in Ukraine

**DOI:** 10.3390/foods14132249

**Published:** 2025-06-25

**Authors:** Yuriy Danko, Viktoriya Kolodiazhna, Olena Nifatova, Anhelina Halynska, Kseniia Bliumska-Danko, Oleksandr Kovbasa, Liuba Turchyn

**Affiliations:** 1 Economics and Management Department, Sumy National Agrarian University, 160 Herasym Kondratieva Street, 40000 Sumy, Ukraineolena.nifatova@ub.edu (O.N.); kseniia.bliumska-danko@universite-paris-saclay.fr (K.B.-D.);; 2 Barcelona Economic Analysis Team, University of Barcelona, 696 Avinguda Diagonal, 08034 Barcelona, Spain; 3 Research Networks, Innovation, Territories and Globalisation Laboratory, Université Paris-Saclay, 54 Boulevard Desgranges, 92330 Sceaux, France; 4 Department of Marketing, Trade and Services, University of West Bohemia, 3-Jižní Předměstí, 30100 Pilsen, Czech Republic; turchyn@fek.zcu.cz

**Keywords:** agricultural raw materials, artisanal food, organic food, farms, sustainable development, marketing

## Abstract

This study explores the marketing potential of artisanal agri-food products in Ukraine by examining their structural, regional, and consumer dimensions. Amid growing interest in sustainable and locally rooted food systems, the research addresses how artisanal production can evolve from informal crafts to market-recognized value. This study is based on an analysis of official statistical data and an analysis of registered artisanal food producers in specific areas in Ukraine. It emphasizes the role of household-based agriculture in securing raw materials, the impact of cultural–tourism infrastructure on product visibility, and the benefits of self-sufficiency in raw material sourcing for product differentiation. The findings reveal that regions with stronger household production and cultural engagement demonstrate higher activity in the artisanal food sector. This study concludes that artisanal food production offers a viable pathway for regional development, rural resilience, and the strengthening of local food identity.

## 1. Introduction

Artisanal food production in Ukraine has significant potential for growth and development. With its rich agricultural resources, favorable climatic conditions, and long-standing agricultural traditions, Ukraine has an ideal foundation for developing the artisanal food industry.

The development of artisanal food production plays a significant role in contributing to various aspects of the economy and culture, as well as the development of society. Ukraine has a rich heritage of traditional artisanal products, including embroidery, pottery, wood carving, weaving, and folk art [1,2]. Artisanal production development helps preserve these conventional techniques, skills, and patterns, ensuring intergenerational transmission and maintaining cultural identity [1,3]. Artisanal production also provides economic growth and employment opportunities, especially in rural areas [4,5]. It supports local artisans, small entrepreneurs, and micro-entrepreneurs, enabling them to generate income and contribute to the local economy [6]. By promoting artisanal products on domestic and international markets, Ukraine can attract tourists and increase revenues from cultural tourism [7]. By encouraging and supporting artisanal activities, Ukraine can help retain people in rural areas, preventing migration to urban centers and promoting balanced regional development [8]. Artisanal production often employs sustainable practices using local materials and traditional production methods [5,9,10]. Artisanal workshops, festivals, and markets serve as cultural exchange, skill-sharing, and networking platforms. This strengthens community ties, promotes cultural diversity, and increases social well-being [10]. Artisanal production encourages artistic expression and innovation by allowing artisans to create unique and distinctive products. [11]. By combining traditional techniques with modern designs, artisans can adapt their products to changing market trends and consumer preferences, appealing to a broader audience both domestically and internationally [2,12]. Ukrainian artisanal foods have the potential to be exported, contributing to the country’s international trade. Ukrainian artisans can enter global markets and increase foreign exchange earnings by focusing on quality craftsmanship, design innovation, and market-oriented strategies [13].

Beyond its economic impact, artisanal food production uniquely intersects with consumer behavior. According to consumer behavior theories, purchasing decisions in the artisanal food market are shaped by a complex set of intrinsic and extrinsic product attributes, including local identity, perceived quality, sustainability considerations, and certification schemes [14,15]. Consumers are increasingly motivated by food safety, environmental concerns, and community engagement. Studies show that information about carbon emissions positively influences purchasing behavior, while certification labels such as PDO and PGI enhance perceptions of product quality and sustainability [15]. Furthermore, the “place attachment” theory suggests that consumers form emotional bonds with local and regional products, associating them with authenticity, cultural identity, and support for rural communities [16].

Social dynamics also play a critical role in shaping consumer choices. Social pressure, family composition (e.g., the presence of children at the table), and community expectations influence decisions to buy artisanal products, aligning with theories of green consumer behavior [17]. In the artisanal food sector, these factors highlight the importance of sustainability communication, storytelling, and transparency regarding production methods and environmental impact.

Artisanal production often relies on sustainable practices, such as using local materials, reducing carbon emissions, and employing traditional methods [18]. These contribute to environmental preservation and align with broader consumer trends toward ethical consumption, eco-friendly packaging, and responsible sourcing.

In general, the development of artisanal food production in Ukraine is relevant because it protects cultural heritage, stimulates economic growth, empowers communities, promotes sustainable development, and stimulates creativity and innovation. It has the potential to benefit both craftspeople and the country by integrating traditional skills with modern market requirements. By combining consumer behavior insights, such as the perception of authenticity, environmental responsibility, and community values, artisanal food producers can better meet market demands and contribute to a more sustainable and inclusive agri-food system.

Based on the generalized insights, we argue that the development of artisanal food production in Ukraine should be interpreted through contemporary consumer behavior theories. Specifically, Social Identity Theory [19,20] helps explain how consumers perceive the purchase of artisanal products as an expression of solidarity with local farmers, support for cultural heritage, and alignment with shared values. Place Attachment Theory [19] highlights the emotional connection between people and specific locations, which, in the context of Ukraine’s artisanal sector, fosters loyalty to products that reflect local traditions, raw materials, and craftsmanship. Meanwhile, Green Consumer Behavior Theory [21] underscores the growing relevance of environmental awareness, as many consumers associate artisanal products with natural ingredients, low carbon footprints, and sustainability. These theoretical perspectives provide a multidimensional understanding of the motivations driving artisanal food production and consumption in Ukraine, extending the analysis beyond purely economic factors and emphasizing the importance of cultural, social, and environmental dimensions.

This study aimed to investigate the current state of production of artisanal agricultural foods in Ukraine, outline the main promising vectors of development of the artisanal food market, and investigate the existing production and raw material potential of this branch of agricultural food production.

## 2. Materials and Methods

To achieve the aims of this study, the following research assumptions (RAs) were formulated:

**RA1.** The development of the artisanal agri-food market in Ukraine exhibits significant regional disparities. It focuses on several key product categories shaped by both natural conditions and the availability of market infrastructure.

**RA2.** The structural diversity and activity level of small farms and household producers are among the contextual characteristics of regions where artisanal agri-food production is developing.

**RA3.** Family-based production is a key source of raw materials for artisanal agri-food products.

These research assumptions formed the basis of the analysis and guided the selection of content analysis methods in this study.

This study did not examine all Ukrainian producers of artisanal food and agricultural foods in rural areas, but only those present in the Ukrainian information space and registered as small business entities—small farms and micro farms. Their legal addresses and the types of artisanal agricultural foods they produce in the domestic market were identified from open information sources [22]. Artisanal production is a continuation of agricultural food production and includes processing agricultural raw materials, the production of finished products, their storage, and their sale, among others. Producers of artisanal and agricultural foods are also engaged in purchasing inputs. This study found that a feature of producing artisanal agricultural foods is the availability of related services to consumers of such products. Such services include excursions to existing cultural and historical monuments and museums, production facilities of the enterprise, tasting of finished artisanal foods, accommodation in local hotels, master classes, and children’s entertainment. The study of services associated with the production of artisanal agricultural foods is not the subject of this study, as they do not directly affect the process of artisanal food production and development prospects, but are relevant cultural and historical “packaging”, i.e., the brand of such products, and require separate scientific research.

The methods used in this study are based on a dialectical approach to the study of economic phenomena, namely, general methods (analysis and synthesis using abstraction and generalization methods used to study the current state and identify the potential for the development of the artisanal agricultural foods market and to formulate conclusions) and special economic methods (analysis of dynamic series to determine the dynamics of changes, comparison to identify trends, etc.). The design of this study is exploratory and descriptive. Instead of applying statistical methods to test hypotheses, we formulated research assumptions that served as guiding themes for the analysis. The empirical base included open-access registries of small producers, regional agricultural reports, and official datasets published by government agencies. Our goal was to identify observable patterns in Ukraine’s territorial distribution and typological structure of artisanal food production. The analysis was based on frequency counts, regional comparisons, and a descriptive interpretation of trends.

The materials used in this study were obtained mainly from sources that published information on the state of production of certain types of agricultural foods, namely, the State Statistics Service of Ukraine. This study used information from 2010 to 2021 (the State Statistics Service of Ukraine has not yet published official data for 2022 at the time of this study).

The methodological explanations in the State Statistics Service of Ukraine regarding its officially published data were considered when analyzing the state of agricultural food production in Ukraine based on different categories of producers (especially households).

1. The current state of the market of artisanal agricultural foods in Ukraine was studied. Descriptive and graphical methods were used to examine the development of the artisanal agricultural food market in Ukraine. This study relied on data from an open-access database [22], focusing on officially registered business entities. Additionally, a qualitative analysis of available company profiles and reported activities was conducted to identify key patterns, regional characteristics, and product types.
2. The main types of artisanal agricultural foods in Ukraine were identified, individual industries and production areas were selected and ranked, and Ukrainian regions were ranked based on the production of certain artisanal foods. The share of each area in the total sales of artisanal agricultural foods was determined based on the number of producers of artisanal products and the types of products in a particular region.
3. The potential for developing the artisanal and agricultural foods market in Ukraine was determined. Descriptive and graphical research methods were used to analyze the potential for expanding the market for artisanal and agricultural foods in Ukraine. The information base for this analysis was data from the State Statistics Service of Ukraine in the context of livestock and crop production, as well as the relevant statistical information necessary for the study of aggregate groupings. Information available on groupings of farms by number of farm animals as of 1 January 2022 was used. Additionally, the following information was used: ratio of farms to households; sales of farm animals for slaughter and milk production by households based on region, as a percentage of the total; and ratio of the volume of produced products to purchased products (fruits, berries, and grapes, including canned and dried products) by households in 2021, as a percentage of the annual balance of products by region.

## 3. Results and Discussion

Artisanal production creates goods, including food, using traditional methods and techniques. It emphasizes using high-quality ingredients, attention to detail, and a hands-on approach, often involving skilled artisans and small producers [23]. Artisanal producers take pride in their craftsmanship, creating unique and distinctive products distinguished by taste, quality, and authenticity [7,24,25].

The essence of artisanal agricultural food production is a unique combination of traditional local practices, attention to detail, and a focus on producing high-quality, artisanal foods. Artisanal production in agriculture covers various aspects that distinguish it from conventional methods of mass production [12]. It includes the following:

Small-scale and localized: Artisanal agricultural food production usually occurs on a small scale, often within local communities or family farms [25,26]. This allows for personalized attention and care throughout the production process. Artisanal producers prioritize quality over quantity, emphasizing local ingredients and sustainable farming practices [26].

Traditional farming methods: Artisanal agriculture often relies on conventional farming methods passed down from generation to generation. These methods usually include organic or regenerative practices such as crop rotation, planting companion plants, and natural pest control. By respecting the land and working in harmony with nature, artisanal producers strive to maintain soil fertility and ecosystem health [27].

Handmade: Artisanal production involves hands-on methods and manual labor. From planting and harvesting to processing and packaging, artisans pay close attention to every step of the production process. Manual harvesting, careful quality checks, and traditional preservation methods are standard practices in artisanal agriculture [28].

Quality and taste: Artisanal producers prioritize the quality and taste of their agricultural foods [29]. By selecting specific crop varieties known for their flavor and nutritional value and allowing fruits, vegetables, or grains to mature fully before harvest, artisanal producers aim to improve the flavor profile and overall sensory experience of their products.

Traceability and transparency: Artisanal production often emphasizes traceability and transparency. Consumers are increasingly interested in knowing where their food comes from, how it was grown, and who produced it. Artisanal producers provide this information by directly linking consumers and the farmers or artisans responsible for the agricultural foods [27,29,30].

Cultural heritage: Artisanal agriculture often reflects cultural heritage and regional traditions. It preserves local food cultures, traditional recipes, and farming methods specific to a particular area. Artisanal producers take pride in preserving and promoting these cultural aspects, contributing to the region’s culinary diversity and cultural identity [7,26,31].

Sustainable and ethical practices: Artisanal agricultural food production prioritizes sustainability and ethical practices. This includes minimizing synthetic fertilizers, pesticides, and genetically modified organisms. Artisanal producers often prioritize animal welfare and biodiversity protection by integrating agroecological principles into farming [32].

Artisanal agricultural food production embodies a commitment to traditional farming methods, quality over quantity, and close ties to local communities. It promotes sustainable development, preserves cultural heritage, and offers consumers an authentic and distinctive food experience. Artisanal agriculture celebrates the art of farming and serves as an alternative to industrial agriculture, emphasizing the values of craftsmanship, environmental stewardship, and cultural heritage.

There is a discussion in some publications about whether the raw materials from which artisanal agricultural foods are produced should be organic [32,33]. It should be noted, however, that the decision to produce artisanal agricultural foods from organic raw materials depends on various factors, including the goals and values of producers, consumer preferences, and applicable legislation. Although there are certain advantages to using organic raw materials in artisanal agriculture, it is not necessarily required [23].

Many artisanal farmers choose organic raw materials because they are committed to sustainable and environmentally friendly practices [34]. Organic farming promotes the use of natural fertilizers, rejecting synthetic pesticides and genetically modified organisms (GMOs), and prioritizes soil health and biodiversity [35]. Producing artisanal agricultural foods from organic raw materials supports these principles [29]. At the same time, consumers looking for artisanal farm foods often value natural and organic ingredients. The use of organic raw materials may attract this segment of consumers, as they may prefer products that are free of synthetic chemicals and produced in an environmentally responsible manner. Meeting consumer demand for organic foods can be a market advantage for artisanal farmers [36]. Some producers believe that organic raw materials contribute to the quality and flavor of artisanal agricultural foods. They argue that organically grown ingredients can have a more delicate flavor and higher nutritional value, reflecting the natural conditions under which they were grown. Artisanal producers who prioritize taste and quality may choose organic raw materials for their unique characteristics [37].

Some regions have specific requirements for certification and labeling of organic foods. If artisanal agricultural producers want to label their products as organic, they must comply with the relevant regulations, including certified organic raw materials. Organic certification can provide trust and confidence to consumers looking for organic foods [33].

Artisanal agriculture often emphasizes sustainable practices and minimizing environmental impact [6]. While organic food production can contribute to these goals, it is not the only approach. Some artisanal producers may adopt alternative sustainable practices, such as regenerative agriculture, agroecology, or permaculture, prioritizing soil health, biodiversity, and resource conservation without seeking organic certification [27].

Ultimately, the decision to use organic raw materials in artisanal agriculture production depends on a combination of factors, including producer values, consumer preferences, market demand, and economic viability. While organic food production can offer sustainability and consumer appeal advantages, it is not required for all artisanal agricultural foods [2,11]. Manufacturers should carefully evaluate their circumstances and make informed choices appropriate for their goals and target market. Thus, organic and artisanal agricultural foods are two concepts that focus on different aspects of food production (Table 1).

Although Table 1 outlines the primary differences between organic and artisanal agricultural foods, it is important to acknowledge where these categories intersect or conflict, particularly regarding consumer perception and market positioning. For instance, organic foods are primarily defined by compliance with certification standards, environmental sustainability, and health-conscious agricultural practices. In contrast, artisanal foods emphasize traditional methods, craftsmanship, and unique sensory experiences, often without formal certification.

However, consumer expectations frequently overlap: many associate artisanal products with naturalness, sustainability, and health benefits—characteristics typically attributed to organic products. This perception can lead to market confusion, where artisanal products are seen as inherently “organic,” even if they do not meet formal certification standards. Conversely, certified organic products may lack the cultural narrative and perceived authenticity that attract artisanal food consumers.

Moreover, from a market strategy perspective, both segments cater to niche markets willing to pay a premium for quality and value-driven attributes. However, certification costs in the organic sector may limit small-scale artisanal producers from obtaining organic labels, creating a barrier to entry. Artisanal producers often rely on trust-based marketing, emphasizing local origin, storytelling, and direct consumer relationships, while organic products depend on third-party certification to signal credibility. The most common types of artisanal food products are artisanal cheeses, wines, bread and pastries, small-batch jams and preserves, local honey, sausages and smoked meats, and specialized condiments and sauces [12,30]. These examples represent only a fraction of the diverse range of artisanal foods. Artisanal foods encompass various culinary creations, each reflecting the artisans’ passion, experience, and dedication.

**Figure 1:**
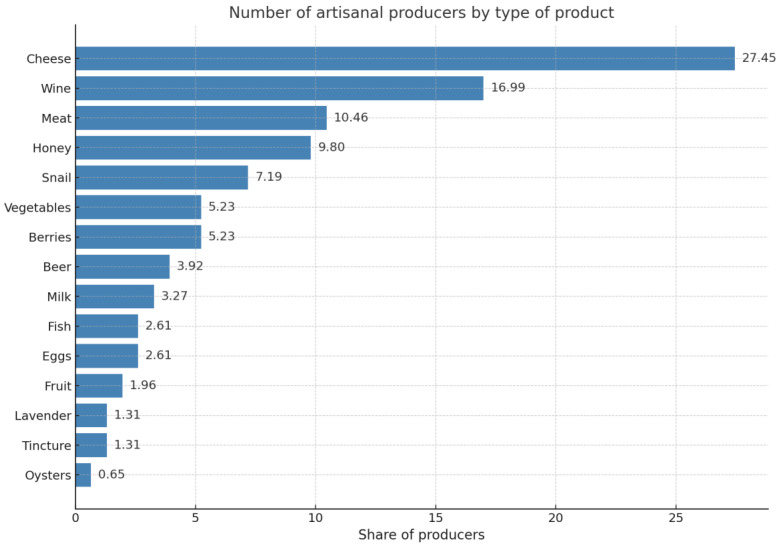
Number of artisanal producers by type of product, as a % of the total.

In our study, we focused on analyzing artisanal agricultural producers who produce their products under their own brand [36,38]. During this study, we identified and analyzed 153 registered business entities that produce artisanal foods using agricultural raw materials from their own production. We also conducted a content analysis of their activities from open sources of information and identified the main types of artisanal foods they produce (Figure 1).

The study of artisanal producers was conducted by region in Ukraine: southern Ukraine (Odesa, Mykolaiv, Kherson regions), the Carpathian region (Zakarpattia, Ivano-Frankivsk, Chernivtsi regions), western Ukraine (Lviv, Ternopil, Khmelnytskyi, Volyn, Rivne, Vinnytsia, Zhytomyr regions), the center and north (Kyiv, Cherkasy, Poltava, Kirovohrad, Chernihiv, Sumy regions), and eastern Ukraine (Kharkiv, Donetsk, Luhansk, Dnipro, Zaporizhzhia regions).

**Figure 2:**
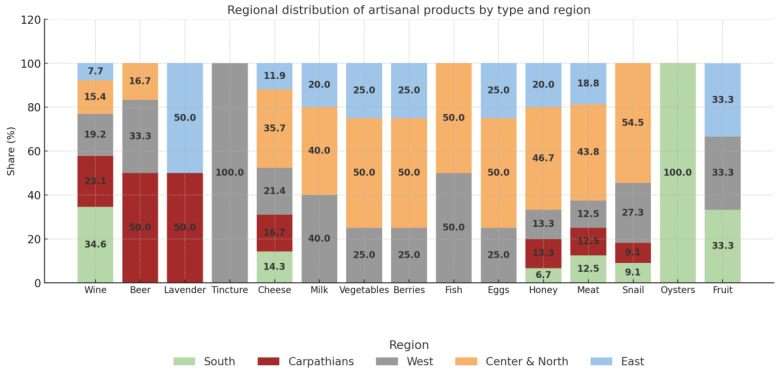
Production of artisanal foods from agricultural raw materials in Ukraine by region and type of product, as a % of the total production of a particular type of product.

This division is appropriate, as the production resources used by agricultural raw material producers vary considerably depending on their geographical location, and, therefore, the types of their artisanal foods also differ. We present the production of artisanal foods (with the share of individual types) in Ukraine by region in Figure 2.

For example, the south of Ukraine has significant potential to produce artisanal agricultural foods due to its favorable climate, fertile soils, and diverse agricultural resources. This diversity allows the production of a variety of artisanal agricultural foods, such as artisanal wines (34.6% of the total), artisanal cheeses (14.3% of the total), artisanal honey products (6.7%), artisanal meat products (12.5%), artisanal snail products (9.1%), and artisanal oyster products (100%). The 100% value for the southern region in the production of artisanal oysters is explained by the absence of other registered producers in other regions of Ukraine in the available open sources at the time of this study. It does not indicate absolute dominance at the national level but rather reflects the territorial concentration of production, driven by natural factors (proximity to the sea) and market factors (availability of specialized infrastructure and logistics).

The natural beauty, historical monuments, and rural landscapes of southern Ukraine make it an attractive tourist destination. Agritourism, which involves immersing visitors in agriculture, allows artisanal agricultural producers to showcase their products and communicate with tourists. This can include farm visits, tastings, masterclasses, and selling artisanal agricultural foods directly to visitors.

The Carpathian region of Ukraine has a huge potential for the production of artisanal agricultural foods due to its unique natural environment, rich biodiversity, traditional farming methods, and growing interest in organic and authentic food [39], with stunning landscapes, including mountains, valleys, forests, and rivers. The available biodiversity is also used in producing artisanal agricultural foods, such as unique honey varieties, herbal teas, and feed and specialty products derived from local plant species. The pristine environment, free from industrial pollution, offers opportunities for organic and sustainable farming, which aligns with the principles of artisanal agriculture.

The following types of artisanal agricultural foods are produced in the Carpathian region: artisanal wines, 23.1% of the total volume; artisanal beers, 50.0%; artisanal cheeses, 16.7%; artisanal honey products, 13.3%; artisanal meat products, 12.5%; and artisanal snail products, 9.1%.

The Carpathian region is rich in cultural heritage, with vibrant traditions, folklore, and unique culinary customs. Artisanal agricultural foods produced in this region can showcase distinctive flavors, ingredients, and traditional recipes reflecting local cultural identity. The region’s popularity as a tourist destination, especially for nature lovers and those seeking authentic experiences, opens opportunities for artisanal producers to interact with visitors, promote their products, and contribute to the local economy through agritourism [40]. The Carpathian region is also home to several products granted protected geographical indication (PGI) status, such as Carpathian honey and Carpathian sheep cheese (brynza). These designations recognize the region’s specific characteristics, traditional production methods, and reputation for quality. Geographical indication status can provide a competitive advantage to artisanal agricultural foods from the Carpathian region, guaranteeing their authenticity and protecting them from counterfeiting.

Western Ukraine has significant potential to produce artisanal agricultural foods due to its fertile land, diverse agricultural resources, cultural heritage, and growing interest in artisanal and local foods. Western Ukraine boasts a diverse agricultural sector, with a wide range of crops, livestock, and specialty products. The region’s abundant raw materials and ingredients offer great opportunities for artisanal producers to create unique and distinctive products [41].

Almost all types of artisanal foods are produced in this region. These include 19.2% of the total volume of artisanal wines, 33.3% of artisanal beers, 100% of artisanal tinctures, 21.4% of artisanal cheeses, 40% of artisanal dairy products, 25% of artisanal vegetables; 25% of artisanal foods from berries and vegetables, 50% of artisanal foods from fish, 25% of artisanal foods from eggs, 13.3% of artisanal foods from honey, 12.5% of artisanal meat products, 27.3% of artisanal foods from snails, and 33.3% of artisanal foods from fruits.

The center and north of Ukraine have significant potential for producing artisanal agricultural foods due to their favorable geographical locations, diverse agrarian resources, market access, and growing consumer demand for authentic and high-quality food products.

Ukraine’s central and northern regions benefit from proximity to major urban centers, transport networks, and export routes. This favorable location provides easy access to domestic and international markets, offering artisanal farmers greater opportunities to sell their products.

These regions have diverse agricultural resources, including fertile soils, large areas of arable land, and favorable climatic conditions. These factors make the regions suitable for various agricultural activities, such as grain, fruit, and vegetable production, dairy and poultry farming, and beekeeping. Artisanal agricultural producers can use these resources to create artisanal and specialty products.

The region produces 15.4% of the total volume of artisanal wines, 16.7% of artisanal beers, 50.0% of artisanal lavender products, 35.7% of artisanal cheeses, 40.0% of artisanal dairy products, 50.0% of artisanal vegetables, 50.0% of artisanal foods from berries, 50.0% of artisanal foods from fish, 25.0% of artisanal foods from eggs, 46.7% of artisanal foods from honey, 43.8% of artisanal foods from meat, 54.5% of artisanal foods from snails, and 33.3% of artisanal foods from fruits.

Different regions in the center and north of Ukraine are known for their specific agricultural specializations. For example, Kyiv and its surroundings are famous for traditional dairy products and honey, and Poltava is famous for producing artisanal honey products. Artisanal agricultural producers can take advantage of these regional specialties and promote them as distinctive products that showcase the area’s unique characteristics.

These regions also have well-developed agricultural infrastructure, including processing facilities, transport networks, and market access. This infrastructure supports the production, processing, and distribution of artisanal agricultural foods. Farmers’ markets, specialty food stores, online platforms, and cooperation with local restaurants and retailers provide artisanal producers with opportunities to engage consumers and showcase their products.

The eastern Ukraine region also has significant potential to produce artisanal agricultural foods due to its vast agricultural land, diverse climatic conditions, and market access. However, it should be noted that in recent years, the region has faced force majeure circumstances caused by Russia’s military aggression.

The eastern Ukraine region produces 7.7% of artisanal wines, 50.0% of artisanal lavender products, 11.9% of artisanal cheeses, 20.0% of artisanal dairy products, 25.0% of artisanal vegetables and berries, 50.0% of artisanal eggs, 20.0% of artisanal honey, 18.8% of artisanal meat products, and 33.3% of artisanal fruit products.

Eastern Ukraine has various climatic conditions, from temperate to continental. This diversity allows for the cultivation of different crops and the production of a variety of artisanal agricultural foods. For example, the southern part of eastern Ukraine has a warmer climate, making it suitable for vineyards and orchards, while the northern part can support grain production and livestock farming. Artisanal producers can use these climatic variations to specialize in products specific to a particular region.

Despite the current military challenges, eastern Ukraine has great artisanal agricultural food production potential. Using its agricultural resources, climate diversity, cultural heritage, and market access, the region can develop a dynamic artisanal agriculture sector that will contribute to economic growth, preserve traditions, and offer consumers unique and high-quality food products.

For example, the southern region of Ukraine produces 13.16% of the total volume of artisanal foods in Ukraine and provides 16.56% of the total number of services associated with artisanal foods; 13.73% of artisanal foods are produced and 14.17% of services are provided in the Carpathian region; 22.88% of the total volume of artisanal foods is produced and 20.65% of related services are provided in western Ukraine; 35.99% of the total volume of artisanal foods and 34.14% of the total related services are provided in the center and northern region of Ukraine; and 14.38% of artisanal foods are produced and 14.17% of associated services are provided by artisanal food manufacturers in eastern Ukraine.

It should also be noted that the development of the artisanal market is influenced by the establishment of cooperation between artisanal producers, farmers, local communities, and agricultural organizations. Sharing knowledge, best practices, and resources can help improve the quality, productivity, and competitiveness of artisanal farm foods; in addition, establishing links with retailers, restaurants, and local food markets can expand market opportunities for artisanal producers in Ukraine and beyond.

**Figure 3:**
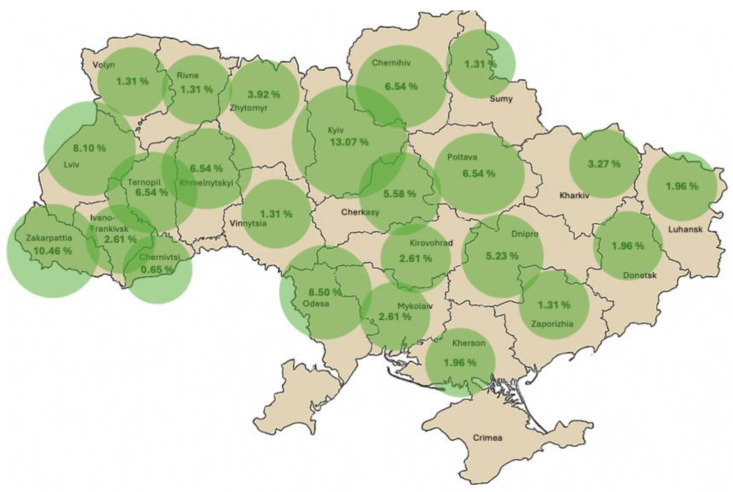
Share of artisanal foods made from agricultural raw materials in Ukrainian regions, as a % of total sales of artisanal foods.

The information on the sale of different types of artisanal foods (by region) in Ukraine is illustrated in Figure 3.

Most of the artisanal agricultural foods are produced and sold in the Kyiv region (13.1% of the total volume of artisanal foods), Zakarpattia region (10.5% of the total volume of artisanal foods), and Odesa region (8.5% of the total volume), while the lowest sales of artisanal agricultural foods are in Vinnytsia, Zaporizhzhia, Rivne, and Sumy regions (1.3% each), as well as Dnipro city and the Chernivtsi region (0.7% of the total volume).

Once again, the data confirm that access to domestic sales channels is extremely important for developing artisanal agricultural producers. Kyiv and the Kyiv region have the most opportunities for effective sales of artisanal foods, as there is a well-developed infrastructure of restaurants and cafes, as well as large retail chains, through which producers can effectively sell their products if they communicate sufficiently to form wholesale batches. We also observe large sales of artisanal foods in Ukrainian regions with developed cultural and historical infrastructure, tourist routes, and a functioning creative industry (Odesa, Poltava, and Zakarpattia regions).

To study the potential for developing artisanal food production from agricultural raw materials, it is necessary to determine which raw materials are most used by producers and to investigate their current production volumes.

We found that the most significant demand is for products made from dairy raw materials (artisanal cheeses), grapes (artisanal wines), and animal raw materials, i.e., meat from farm animals (artisanal meat products). The share of artisanal honey products is also significant; thus, it is necessary to determine the production volumes of the following agricultural raw materials: milk, meat from various types of farm animals, grapes, and honey.

It should also be noted that most producers of artisanal agricultural foods are small business entities. These include small and micro farms, farms, and households.

For further analyses, we grouped farms by the number of farm animals (up to 50 heads of cattle, cows, sheep, and goats; up to 100 heads of pigs; up to 4999 heads of poultry) per enterprise as of 1 January 2022 (Table 2).

The results of the analyses showed that the number of animals in the groups of farms is as follows: cattle, 0.9% of the total number of livestock; cows, 2.2%; pigs, 0.5%; sheep and goats, 2.2%; and poultry, 0.1%. The number of farm animals kept by small and micro farms is insignificant, but the percentage of these farms as a ratio of the total number of agricultural farms ranges from 24.4% for the cattle group to 32.4% for the sheep and goats group. Additionally, most farm animals are kept by medium and large farms that do not fall under the definition of artisanal agrarian producers.

Households may become a promising group of artisanal producers [1,4,10,34]. We compared the volumes of agricultural raw materials that farms produce, including farms and families. The results of the analysis are presented in Table 3.

Enterprises produce the following types of farm animal meat: pork, 59.7%; poultry meat, 88.0%; and different types of eggs, 49.8%. In total, farms produce 70.6% of farm animal meat. At the same time, households produce 74.9% of beef and veal meat, 90.2% of lamb and goat meat, 93.6% of rabbit meat, 97.2% of horse meat, 68.2% of different types of milk, 50.2% of all types of eggs, 89.9% of wool, and 99.0% of honey.

**Figure 4:**
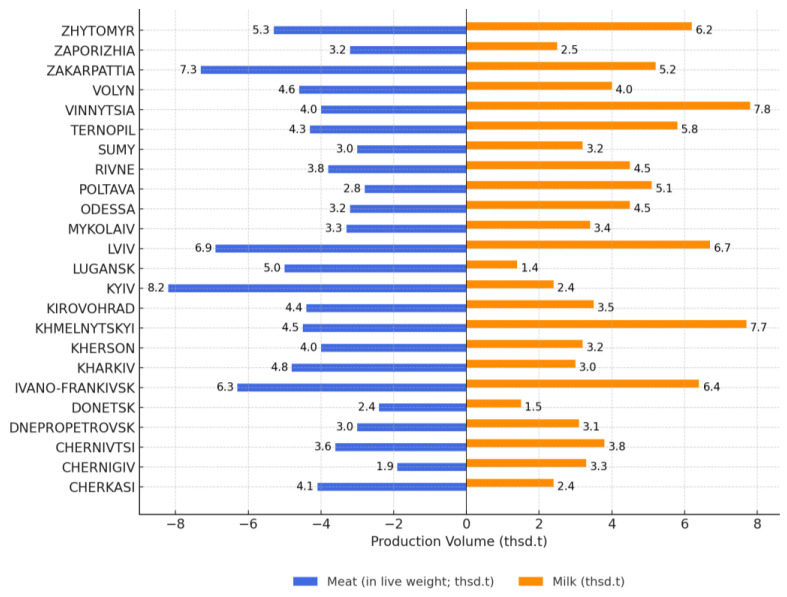
Regional sales of all types of farm animals slaughtered for meat and all types of milk produced by households in Ukraine, as a percentage of total sales.

To more accurately determine the regional potential for the production of agricultural raw materials for artisanal foods in Ukraine, we determined the volume of all types of slaughtered farm animals and all types of milk produced by households as a share of the total volume of sales (Figure 4).

The results of this study show that households in Khmelnytskyi, Ternopil, Lviv, Kyiv, Ivano-Frankivsk, Zakarpattia, Zhytomyr, Volyn, and Vinnytsia regions have the greatest potential for producing agricultural raw materials that can be used for producing agricultural artisanal foods.

For example, in the Khmelnytskyi region, households produce 7.7% of the total volume of milk and 4.5% of the total quantity of farm animal meat (by live weight); Ternopil region produces 5.8% of the milk and 4.3% of the meat; Lviv region produces 6.7% of the milk and 6.9% of the meat, Ivano-Frankivsk region produces 6.4% of the milk and 6.3% of the meat; Zakarpattia region produces 5.2% of the milk and 7.3% of the meat; Zhytomyr region produces 6.2% of the milk and 5.3% of the meat; and Vinnitsa region produces 7.8% of the milk and 4.0% of the meat. These Ukrainian regions are potentially suitable for producing artisanal foods, since they contain households that produce abundant quantities of the required raw materials (meat and dairy products).

**Figure 5:**
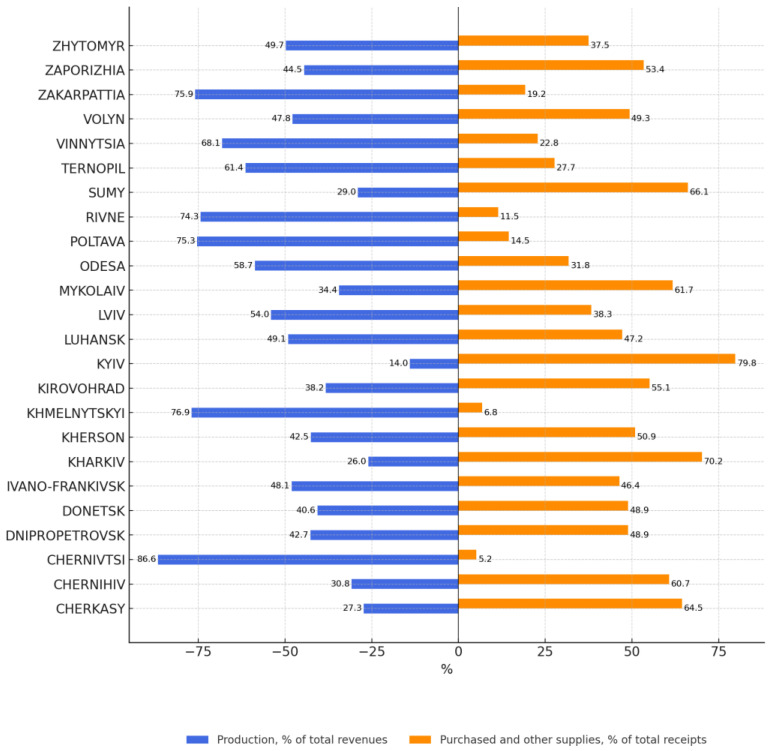
The ratio of the volume of produced products to purchased products (fruits, berries, and grapes, including canned and dried products) in households in 2021, as a percentage of the annual product balance by region in Ukraine.

Similarly, we studied the potential of households in these regions to produce agricultural crop products—fruits, berries, and grapes—that can serve as raw materials for artisanal foods (Figure 5).

The annual balance sheet for products (fruits, berries, and grapes) is generated as follows: the item “Income” consists of the stock at the beginning of the year, the production of fruits, berries, and grapes, as well as purchases and other receipts during the year. The item “Use” consists of the consumption of feed products, wine processing costs, sales costs, storage losses, and consumption costs. The difference between these items is the carryover balance of production for the next year. In our study, the ratio of own production to purchased products is important as it is economically unprofitable for artisanal producers to buy raw materials for processing as opposed to growing their own [42].

The results of this study show that Chernivtsi, Khmelnytskyi, Ternopil, Rivne, Poltava, Odesa, Lviv, Zakarpattia, and Vinnytsia regions have the greatest potential for producing agricultural raw materials (fruits, berries, and grapes) that can be used to produce agricultural artisanal foods.

For example, households in Chernivtsi region produce 86.6% and buy 5.2% of agricultural raw materials (fruits, berries, and grapes), households in Khmelnytskyi region produce 76.9% and buy 6.8%, households in Ternopil region produce 61.4% and buy 27.7%, households in Rivne region produce 74.3% and buy 11.5%, households in Poltava region produce 75.3% and buy 14.5%, households in Lviv region produce 54.0% and buy 38.3%, households in Zakarpattia region produce 75.9% and buy 19.2%, and households in Vinnitsa region produce 68.1% and buy 22.8%. Mostly fruits, berries, and grapes are bought in Chernihiv, Cherkasy, Kharkiv, Sumy, Mykolaiv, Kyiv, and Zaporizhzhia regions. These regions have the lowest potential to produce artisanal foods from these raw materials.

Family-based production as a source of raw materials for artisanal products can significantly reduce costs. In sectors such as cheesemaking, meat processing, and preserving fruits and vegetables, growing raw materials or raising livestock directly allows producers to minimize procurement costs. For example, artisanal cheese producers often use milk from their own or partner farms, reducing logistics expenses and ensuring freshness. In the meat sector, raising pigs or poultry enables producers to control meat quality and avoid the use of antibiotics. For jams, juices, and preserves, seasonal fruits and vegetables from personal or local farms are often used, lowering production costs and enhancing the regional authenticity of the products. The key advantage in this context is the ability to control the entire value chain, both in terms of quality and cost management.

Recent research on branding in the agri-food sector underscores the importance of green positioning as a driver of consumer loyalty and product value. Empirical evidence from Ukrainian supermarkets reveals that consumers are more loyal to eco-labeled brands than regular agri-food products. In one case, loyalty to eco-branded eggs exceeded regular brand loyalty by 3% [43]. This suggests that the credibility of artisanal food production can be significantly enhanced by incorporating elements of green branding and certification, even in highly competitive product categories.

The price premium consumers are willing to pay for artisanal or eco-labelled goods is not uniform. It is influenced by a combination of factors, including consumer loyalty, presence of organic certification, marketing investments in green promotion, and the average income level of the target demographic [44,45].

These insights point to the broader applicability of market segmentation, product storytelling, and strategic branding in the artisanal agri-food sector. By aligning brand identity with consumer expectations around sustainability and authenticity, producers can enhance loyalty and justify higher price points [16]. This is particularly relevant for artisanal food farms that rely on traditional methods, local raw materials, and transparent supply chains, characteristics that resonate with environmentally and ethically conscious consumers.

It is also essential to consider the war-driven shift in the geography of artisanal food production. Several Ukrainian regions historically had strong household production and cultural-tourism potential, such as parts of the south and east, which have been severely affected by military action or remain under occupation. This disruption has led to the temporary or permanent loss of artisanal initiatives in those areas. In contrast, regions in the west and the center have seen an influx of displaced producers, resulting in new patterns of artisanal activity and local concentration.

Moreover, the closure of seaports and the loss of external agri-food markets due to the war have accelerated a reorientation toward domestic consumption. Many producers who previously focused on export or wholesale have begun adapting their products and marketing strategies to meet the needs of local and regional consumers. This inward shift has spurred the emergence of shorter supply chains and more direct producer–consumer relationships, strengthening the role of artisanal food in national food security and rural economies.

## 4. Conclusions

This study highlights the expanding role of artisanal agri-food production in Ukraine as a response to structural and circumstantial pressures. The observed patterns suggest a possible association between the activity of household-based agricultural producers and the development of artisanal food production across regions. These conditions support autonomy in sourcing, enhance authenticity, and foster regional differentiation—factors that significantly contribute to the marketing appeal of artisanal foods.

The artisanal sector has adapted to changing regional conditions and demonstrates resilience through local networks and diversified production. Moreover, the loss of export routes and shifting consumption patterns have pushed many producers to reorient toward domestic markets. Short supply chains and direct marketing gained new significance as traditional distribution chains collapsed or became inaccessible. In this context, artisanal food is no longer a peripheral alternative but a viable strategy for economic recovery, local branding, and supply chain resilience.

Regions combining agricultural self-reliance, cultural embeddedness, and entrepreneurial adaptation, such as Zakarpattia, Lviv, and parts of Central Ukraine, have emerged as key nodes in the evolving artisanal landscape. Their success lies in product quality and the ability to tell a story, connect to place, and engage with communities. These features are crucial for transforming local crafts into competitive market niches. To explain regional differences in the share of artisanal food sales, it is essential to consider the broader economic and political context. For example, regions with a higher concentration of artisanal producers and greater market penetration often coincide with a developed tourism infrastructure, stronger local consumer demand, and more active participation in regional branding or gastronomic promotion initiatives. These factors indirectly contribute to the market visibility and success of small-scale producers. However, due to the limitations of the available data, a detailed econometric assessment of political subsidies or investment flows was not conducted, which may also influence these patterns. Further research is needed to explore these structural factors and their role in shaping regional competitiveness in the artisanal food sector.

Ultimately, artisanal agri-food products in Ukraine offer more than economic potential; they act as symbolic vehicles of place-based identity and social continuity amid disruption. As Ukraine continues to face geopolitical and market challenges, the artisanal sector represents a grassroots-driven model of adaptive food systems that is worth supporting through targeted policy, certification schemes, and inclusion in regional development strategies.

This study is limited by its reliance on secondary data and the absence of primary data collection. The formulated assumptions were not statistically tested but were guided by a thematic analysis of official registries and regional sources. Additionally, this study focuses solely on registered small enterprises and does not account for informal family-based productions, which may also contribute significantly to the development of the artisanal industry. This may lead to an underestimation of the sector’s actual production potential. Future research could focus on exploring informal family-run production units, which would provide a more comprehensive understanding of the marketing potential of the artisanal industry.

Furthermore, this study focused only on food-related enterprises. Investigating other artisanal sectors could also be valuable. Further empirical research, including fieldwork and surveys, would complement these findings.

This study provides insights that extend beyond the Ukrainian context and may inform strategies for supporting artisanal food production in other European countries. The findings highlight the importance of combining household-based production systems, cultural heritage, and local raw material self-sufficiency as sustainable and resilient agri-food system models. These lessons are particularly relevant for regions across Europe that aim to revitalize rural economies, preserve traditional practices, and meet growing consumer demand for authentic, high-quality, and environmentally responsible food products. The case of Ukraine illustrates how artisanal production can serve as a tool for rural resilience and regional branding, contributing to economic diversification and cultural preservation, even under challenging geopolitical circumstances. These insights can guide EU policies, including rural development strategies, green branding initiatives, and funding programs to enhance the visibility and competitiveness of small-scale artisanal producers within the broader European market.

## Figures and Tables

**Table 1:** The main differences between organic and artisanal agricultural foods.

Difference	Description
Organic agricultural products	
Production methods	Organic agricultural products are grown or produced in accordance with specific guidelines and rules set by organic certification bodies. These rules typically prohibit the use of synthetic fertilizers, pesticides, genetically modified organisms (GMOs), and irradiation. Organic farmers favor the use of natural fertilizers, crop rotation, biological pest control, and sustainable farming practices.
Certification	Organic agricultural products can be certified by authorized organizations that confirm that they meet established organic standards. Certification requires adherence to strict rules, periodic inspections, and documentation to ensure compliance. Products labeled as “organic” must meet the criteria set by the certification body.
Focus on the environment	Organic agriculture places a strong emphasis on environmental sustainability and minimizing the impact on ecosystems. It aims to maintain soil health and biodiversity, and conserve natural resources. Organic agriculture strives to work in harmony with nature, reducing dependence on synthetic inputs and promoting ecological balance.
Regulatory and legal frameworks	Organic agricultural production is governed by specific rules and standards set by government agencies or private organizations. These regulations define permitted practices, labeling requirements, and certification guidelines. The certification process involves regular inspections and audits to ensure compliance with organic standards.
Artisanal agricultural products	
Artisanal production	Artisanal agricultural products emphasize traditional, artisanal, or small-scale production methods. Craft producers often use time-honored technologies while preserving traditional knowledge and heritage. They emphasize attention to detail, craftsmanship, and quality of the final product.
Quality and uniqueness	Artisanal agricultural products are known for their special qualities, flavors, and characteristics that result from specific production methods and attention to detail. Artisanal producers can focus on specialty crops, traditional varieties, or unique flavors, emphasizing the expertise and creativity involved in the production process.
Market niche	Artisanal agricultural products cater to a niche market that values authenticity, uniqueness, and support for local producers. Artisanal products often have a strong connection to a particular region, culture, or tradition. Consumers looking for artisanal agricultural products are willing to pay a premium for the craftsmanship, history of the product, and the special experience it offers.
A diverse range of products	Artisanal agricultural products cover a wide range of foods and beverages, including cheeses, meat products, bread, beer, wine, chocolate, etc. The artisanal production approach can be applied to different sectors of agriculture, with a focus on quality, taste, and the human factor involved in the production process.

**Table 2:** Grouping of farms by the number of farm animals as of 1 January 2022.

Products	Number of Livestock per Enterprise	Number of Enterprises		Number of Livestock	
		Units	Percentage of Total Enterprises	Thsd. Heads	Percentage of the Total Number
Cattle	to 50	437	24.4	8.8	0.9
Cows	to 50	536	31.8	9.4	2.2
Pigs	to 100	404	31.2	17.7	0.5
Sheep and goats	to 50	166	32.4	3.7	2.2
Poultry	to 4999	100	31.6	114.5	0.1

**Table 3:** The ratio of agricultural raw material production by farms and households (%).

Product Name	Enterprises	Including Private Farms	Households	Ratio of Enterprises to Households, %		
Meat from farm animals, total, thousand tons	1720.4	86.4	717.9	70.6	/	29.4
beef and veal	77.9	7.2	232.6	25.1	/	74.9
pork	431.9	24.0	292.1	59.7	/	40.3
lamb and goat	1.2	0.0	11.0	9.8	/	90.2
poultry meat	1208.5	55.2	165.0	88.0	/	12.0
rabbit	0.7	0.0	10.2	6.4	/	93.6
horse meat	0.2	0.0	7.0	2.8	/	97.2
All types of milk, thousand tons	2767.7	243.9	5946.2	31.8	/	68.2
All types of eggs, million units	7012.8	144.2	7058.5	49.8	/	50.2
All types of wool, tons	151	42	1346	10.1	/	89.9
Honey, tons	685	121	67,873	1.0	/	99.0

## Data Availability

Data are contained within the article.
